# An empirical assessment of research practices across 163 clinical trials of tumor-bearing companion dogs

**DOI:** 10.1038/s41598-019-48425-5

**Published:** 2019-08-15

**Authors:** Yuan Jin Tan, Ryan J. Crowley, John P. A. Ioannidis

**Affiliations:** 1 Department of Health Research and Policy, Division of Epidemiology, Stanford School of Medicine, Stanford, CA USA; 2 Department of Statistics, Stanford University School of Humanities and Sciences, Stanford, CA USA; 3 Stanford Prevention Research Center, Department of Medicine, and Department of Biomedical Data Science, Stanford University School of Medicine, Stanford, CA USA; 4 Meta-Research Innovation Center at Stanford (METRICS), Stanford University, Stanford, CA USA

**Keywords:** Cancer models, Target validation

## Abstract

Comparative clinical trials of domestic dogs with spontaneously-occurring cancers are increasingly common. Canine cancers are likely more representative of human cancers than induced murine tumors. These trials could bridge murine models and human trials and better prioritize drug candidates. Such investigations also benefit veterinary patients. We aimed to evaluate the design and reporting practices of clinical trials containing ≥2 arms and involving tumor-bearing dogs. 163 trials containing 8552 animals were systematically retrieved from PubMed (searched 1/18/18). Data extracted included sample sizes, response criteria, study design, and outcome reporting. Low sample sizes were prevalent (median n = 33). The median detectable hazard ratio was 0.3 for overall survival and 0.06 for disease progression. Progressive disease thresholds for studies that did not adopt VCOG-RECIST guidelines varied in stringency. Additionally, there was significant underreporting across all Cochrane risk of bias categories. The proportion of studies with unclear reporting ranged from 44% (randomization) to 94% (selective reporting). 72% of studies also failed to define a primary outcome. The present study confirms previous findings that clinical trials in dogs need to be improved, particularly regarding low statistical power and underreporting of design and outcomes.

## Introduction

Comparative oncology trials in dogs have recently been proposed as a novel means of improving human drug discovery^1–4^. Such trials are conducted on companion animals with naturally occurring cancers and are meant to bridge murine models and human clinical trials. While healthy, purpose bred beagles have routinely been used in safety testing of drugs, there is now an emerging argument for expanding the role of canine trials beyond safety alone. Unlike the far more commonly used murine models, dogs with cancer retain their native immune system and tumor microenvironments^2^. The impact of environmental factors on cancer development and progression can also be studied, because, unlike murine models, domestic dogs do not exist in controlled environments^2^. Much as in humans, canine cancers also exhibit periodic recurrence and metastasis^2^. Furthermore, a higher degree of homology exists between canine cancer genes and those in humans than between humans and mice^4^. A comparison of human and canine mammary tumors has also showed similar patterns of up- and down-regulation in various cellular signaling pathways between the two species^5^.

In view of the parallels between human and canine cancer, it has been proposed that comparative trials could help to address the low predictive ability of murine models^1,2,6–8^. Preliminary efficacy results from clinical trials in dogs could be used to guide the design of human clinical trials, thereby increasing efficiency and reducing unnecessary human experimentation. For example, in 1995, a successful trial of liposomal muramyl tripeptide (L-MTP-PE) in dogs with osteosarcoma helped prioritize Phase III pediatric studies of the drug^2,4^. Comparable results between dogs and human patients have since been reported^2,4^. In addition to delivering efficacy insights, trials in dogs have also helped define biomarkers that could be used in subsequent human trials as predictors of treatment efficacy^4^. This potential value to human medicine comes above and beyond the ability for such trials to advance veterinary medicine; for example, when a drug licensed for use in human patients is assessed for efficacy in treating dogs with cancer. A consortium of 22 veterinary centers aiming to conduct multi-center canine trials in oncology, known as the Comparative Oncology Trials Consortium (COTC), was established by the National Cancer Institute in 2003^9^, illustrating the increased prominence of canine trials today.

Nonetheless, despite this great potential, questions still remain. As trials in domestic dogs with cancer have become more common, a crucial issue is whether they are of sufficient quality to obtain reliable results and allow effective translation. High standards for preclinical research are essential^10^, and veterinary clinical trials should be held to a similar standard^11^. These include sound protocols, standardized reporting, and appropriate statistical considerations^10–12^. Some prior work in this field has already started to describe the low sample sizes^13,14^, historical preferences for case studies over prospective veterinary studies^15^, lack of reporting for methodological details including randomization mechanism, blinding, inclusion criteria^14,16–19^, as well as for results such as adverse events^18^. However, as a whole, trials in domestic dogs have still received less emphasis than clinical trials in humans in this regard.

We therefore aimed to build upon the existing body of knowledge and empirically asses the available literature for study design quality and risk of bias. An additional objective was to identify interventions tested and outcomes measured in the included trials. We believe that insights gathered could prioritize objectives for the design of future studies comparing results between dogs, humans, and murine models; and help further establish a rigorous role for clinical trials performed in tumor-bearing dogs as integral to the drug discovery pipeline.

## Methods

### Inclusion and exclusion criteria

Trials enrolling tumor-bearing dogs were identified using the following PubMed search strategy: ((“dogs”[MeSH Terms] OR “dogs”[All Fields] OR “dog”[All Fields]) OR (“canine”[All Fields])) AND (Clinical Trial[ptyp] OR trial* OR randomi*) AND (cancer[sb] OR cancer). The search date was Jan 18, 2018. Studies were excluded if they did not study cancer treatment in companion dogs with naturally occurring disease, did not utilize prospective subject recruitment and data collection, lacked measures of intervention or procedural efficacy, were in a language other than English, or were single arm trials, observational studies (including retrospective studies and case reports), meta-analyses or review articles. Studies investigating all forms of malignancy, including solid tumors and hematological malignancies, were eligible for inclusion. Papers were included regardless of whether they studied small molecule therapies, surgical procedures, biologics, diagnostic procedures, or other non-pharmacological interventions including behavioral therapies. Studies solely reporting safety, pharmacokinetics, or pharmacodynamics were not included. For each article identified in PubMed (n = 2317), eligibility was assessed by a single author (YJT) on the basis of information derived from the abstract or from the full text if necessary. A second independent assessor (RJC) evaluated eligibility in all 2317 articles. JPAI adjudicated any discrepancies between the two authors’ assessments that could not be resolved. The two assessors reached a consensus to include 163 articles.

### Regulatory status of treatment interventions

For clinical trials evaluating treatment efficacy of small molecules (drugs) and biologics, we determined whether the anticancer treatments being evaluated had been licensed by the FDA for use in human patients prior to the veterinary trial. For this purpose, one investigator (YJT) searched Wikipedia and the Drugs@FDA database, as needed, to determine approval status as of December 2018; and to determine the length of time between approval and publication of the canine trial. When unlicensed, YJT searched PubMed and PubChem to find out if any human study using the same drug or biologic had been published before the publication of the respective trial in dogs. In addition, the reference list for included veterinary trials studying unlicensed interventions were searched to determine if they cited any human studies. When tested interventions in veterinary trials included combination of two or more drugs and/or biologic, we examined each one of them separately.

### Sample size and power calculation

For each study, we manually extracted the sample size across the entire study, the sample size per American Cancer Society tumor type^20^ tested per study, and the sample size per intervention arm. In addition, we manually extracted the number of events per arm for both survival and disease progression analyses. The number of events for survival analyses was defined as the number of deaths from any cause, including euthanasia, by the end of the study. For disease progression, this was defined as the number of dogs with disease progression at the end of the study. Only studies that defined progression as tumor growth by a set percentage were included in this analysis. Deaths without disease progression were not considered as a disease progression event. The detectable hazard ratio given the ratio of subjects per arm and number of events was calculated using the Schoenfeld formula^21^, assuming a Type I error rate of 0.05 and power of 0.8. A single assessor (YJT) performed data extraction and analysis.

### Disease progression criteria extraction

To determine if the criteria for therapy response in clinical trials in tumor-bearing dogs had changed over time, we determined if the VCOG RECIST criteria was explicitly named as the criterion used, and extracted the minimum change in tumor size for partial response and progressive disease as defined by the authors in each study. Metrics for size as defined by the authors, such as volume or diameter, were also extracted. A single assessor (YJT) performed data extraction.

### Cochrane risk of bias assessment

Each study underwent a Cochrane risk of bias assessment^22^. In brief, studies were judged for their risk of bias in six categories according to guidance provided for the Cochrane tool: random sequence generation, allocation concealment, blinded participants/personnel, blinded assessment of results, incomplete data, and selective reporting (i.e. trial registration). Studies were assessed to be at high risk of bias, low risk of bias, or to have unclear risk due to insufficient information. Owing to the subjective nature of this assessment, two assessors (YJT and RJC) performed risk of bias analyses independently. JPAI adjudicated any discrepancies between the two authors’ assessments that could not be resolved. The consensus risk of bias assessment for all 163 studies agreed upon by the two assessors is presented in this manuscript.

### Outcome reporting

Outcomes reported as primary outcomes within each paper, as well as outcomes listed in the abstract of each paper were assessed for statistical significance at the level defined by the authors (typically p < 0.05). Studies were assessed for the presence of a defined primary efficacy outcome, a reported abstract efficacy outcome, and ≥1 statistically significant primary or abstract efficacy outcomes. Studies were also assessed for the presence of significant outcomes showing that the experimental active intervention was harmful compared with standard of care or placebo. Studies that did not conduct statistical tests on primary or abstract outcomes were also identified. Untested outcomes were further characterized according to the authors’ qualitative assessments of whether they were favorable, i.e. demonstrated an efficacy benefit versus standard of care or placebo control. A single assessor (YJT) extracted outcome data.

### Data cleaning

Raw data was manually extracted from publications before undergoing a data cleaning and analysis process using R to arrive at data in its presented form. The following R packages were used for cleaning, analysis, and plotting: *readxl*^23^, *stringr*^24^, *tidyr*^25^, *splitstackshape*^26^, *plyr*^27^, *RISmed*^28^, *ggplot2*^29^. Additionally, the *RoB Summary* function was used for creating risk of bias graphs^30^. Tableau was used for additional plotting.

## Results

**Figure 1:**
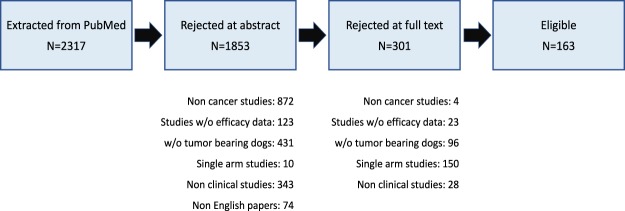
Study flowchart showing publications excluded or included in the analysis.

2317 publications were initially identified from Pubmed. Following exclusion of publications using the criteria detailed in the Methods, 163 papers, each containing a single trial with at least two treatment arms, remained eligible for analysis (Fig. 1). Supplementary Appendix 1 lists the references of these trials. Some key characteristics of the eligible trials appear in Table 1.

**Table 1:** Key characteristics of eligible trials.

**Number of eligible studies**	163
**Range of publication years**	1982–2017
**Study type** ^**#**^	
Singly tested small molecules	82
Singly tested biologics	21
Biologics and/or small molecules in combination	29
Surgical intervention	16
Radiation therapy	27
Dietary regimen/feeding behavior	8
Diagnostic procedure	1
Number of studies per ACS tumor type*	
Bone	32
Digestive	12
Endocrine	5
Eye	3
Genital	10
Leukemia	2
Lymphoma	53
Mammary	28
Mastocytoma	22
Nervous	8
Not reported	23
Oral	19
Other	25
Respiratory	4
Skin	27
Soft tissue	29
Urinary	12

^*^Each trial may have investigated multiple tumor types.

^#^Each trial may have investigated multiple intervention types.

### Regulatory status of treatment interventions

Of the 126 trials that evaluated small molecules (drugs) and biologics tested singly or in combinations, 52 tested drugs or biologics that had been licensed in humans as of December 2018. In all 52 trials, the licensing occurred at least five years prior to the publication of the trial conducted in dogs. The median time between human licensing and publication of the veterinary trial was 26.5 years (IQR 18–38.25). Of the 74 trials assessing drugs, biologics, or both, that had not been previously approved by the FDA for use in humans; 59 solely tested non-FDA licensed interventions, while 15 investigated a mix of licensed and unlicensed interventions. 7 of the 74 interventions were licensed in humans after trials in dogs. In 34 of the remaining 67, the unlicensed drugs or biologics had previously been used in human studies that were published before the publication of the respective veterinary trial. 37 trials tested interventions that had never been studied in humans and 3 trials investigated a mix of previously tested and untested interventions.

### Sample sizes and power

**Figure 2:**
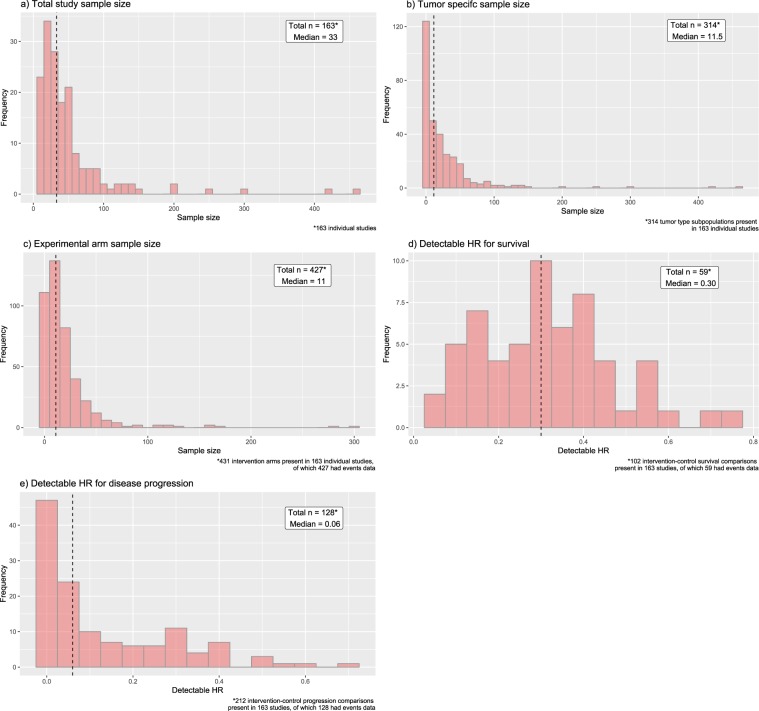
Histograms showing distribution of (**a**) total study sample size, (**b**) sample size by American Cancer Society tumor type^20^, (**c**) sample size by intervention arm, and distributions of detectable hazard ratios (HRs) for (**d**) survival and (**e**) disease progression. The median for each distribution is annotated and represented by the vertical dashed lines.

The sample size distributions were heavily right skewed with the majority of studies having low sample sizes that severely limit their detectable hazard ratios (Fig. 2a–c). The medians for total study sample size, sample size by tumor type, and by intervention arm were 33(IQR 19.5–54), 11.5 (IQR 2–32), and 11 (IQR 5–24.5) respectively. The detectable hazard ratios (HRs) given the number of events reported in these clinical trials in dogs were extremely low as well (Fig. 2d,e). The median detectable HR was 0.3 (IQR 0.18–0.40) for overall survival, and 0.06 (IQR 0.004–0.24) for disease progression. Only 2 trials would have 80% power to detect a HR of 0.7 for overall survival and only 1 trial would have 80% power to detect a HR of 0.7 for disease progression. Among the 59 trials solely evaluating drugs or biologics not previously licensed in humans, medians for total study sample size, sample size by tumor type, and by intervention arm were 42 (IQR 23–70.5), 11 (IQR 2–35.5), and 12 (IQR 4–27) respectively.

### Changes in response criteria over time

In 2010 and 2013, the Veterinary Cooperative Oncology Group (VCOG) published consensus documents describing the canine response evaluation criteria for peripheral nodal lymphoma and for solid tumors (cRECIST v1.1) respectively^31,32^. These guidelines were based on the human RECIST guidelines, and introduced a standardized framework which require lesions to be measured with standardized cutoffs defined for progressive disease, partial response, complete response, and stable disease^33,34^. cRECIST defines partial response as >30% shrinkage in mean sum longest diameter for all target lesions compared with baseline; progressive disease is defined as >20% growth in mean sum longest diameter for all target lesions or appearance of new lesions^32^.

Of the 112 trials that measured disease progression as an outcome, 94 clearly defined an objective measurement criterion. Of these, only 30 explicitly named cRECIST guidelines as the criterion used. The majority of the remaining trials (57/64) were published before the VCOG cRECIST guidelines became available. The 64 trials that did not cite cRECIST as the criterion measured progression as either the appearance of new lesions (27/64) or through changes in tumor size (37/64). Definitions of tumor size varied. The majority of these trials (29/38) defined tumor size as volume or product of diameters. Only 1 trial defined size as diameter. 7 of the 38 trials did not clearly define the metric used to assess tumor size.

Some trials that did not explicitly cite cRECIST guidelines differ from the cRECIST criteria in terms of stringency. All 27 trials that defined disease progression as appearance of new lesions, and the singular trial that defined progression according to changes in tumor diameter implemented thresholds that were equivalent to cRECIST. For the 29 trials that defined tumor size by volume, a 50% reduction in tumor volume is equivalent to a 30% reduction in diameter for partial response. Likewise, a 44% growth in volume is equivalent to a 20% growth in diameter for progressive disease. For partial response, the majority of these trials (28/29) used a 50% reduction in volume and hence have equivalent stringency to cRECIST. However, significant variation existed in the percent growth definition for progressive disease. 14 of the 29 trials used thresholds at 50%, representing a threshold that is less stringent. 7 trials used thresholds at 25%, representing more stringent thresholds. In addition, a further 7 used a more stringent criteria by considering any tumor shrinkage less than partial response as no response and did not make a distinction between no response and progressive disease.

### Risk of bias assessment

**Figure 3:**
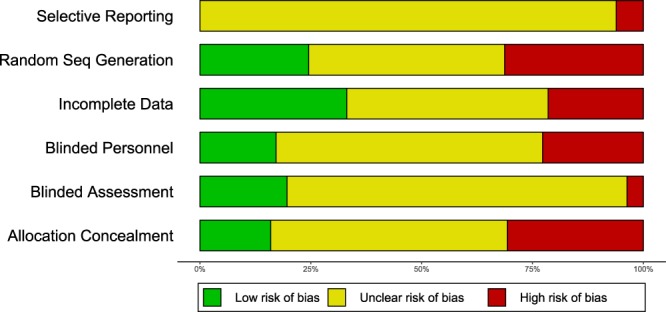
Cochrane risk of bias assessment for canine trials. Shown are the 6 Cochrane tool categories, with risk evaluation color coded as follows: high risk (red), low risk (green), unclear reporting (yellow)

We identified a high degree of unreported experimental details in the included veterinary clinical trials. This underreporting was prevalent in all categories of the Cochrane risk of bias tool, with the Selective Reporting, Blinded Assessment, and Blinded Participants/Personnel categories possessing the highest share at 94% (153/163), 77% (125/163), and 60% (98/163) not reported respectively (Fig. 3). None of the trials reported pre-registered protocols.

### Outcome reporting

**Figure 4:**
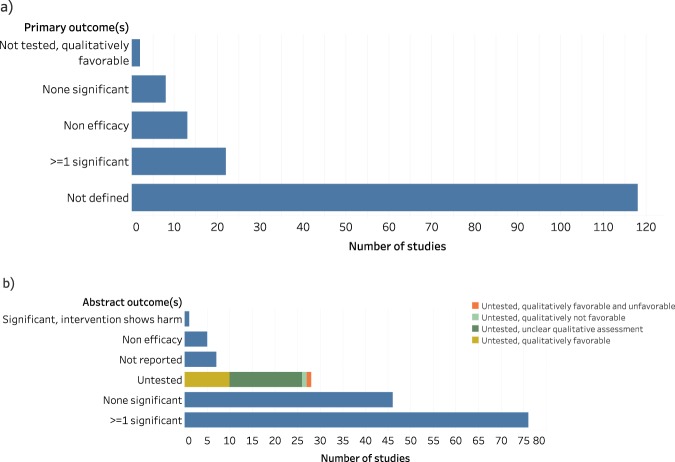
Characterization of primary outcomes (**a**) and outcomes found in the abstract (**b**) for canine trials.

Similarly, there was a high level of underreporting for outcomes as well. 72% (118/163) of studies failed to define a primary outcome (Fig. 4a). In contrast, only 4.3% (7/163) of studies failed to report outcomes in the abstract (Fig. 4b). 13.5% (22/163) of papers contained at least 1 statistically significant primary outcome, and 5% (8/163) of papers contained no significant primary outcomes. For outcomes reported in the abstract, these proportions were 47% (76/163) for statistically significant and 28% (46/163) for none significant. Only 1.2% (2/163) of primary outcomes were not statistically tested while 17% (28/163) of outcomes listed in the abstract were not tested.

## Discussion

Overall, our systematic review of 163 clinical trials enrolling tumor-bearing dogs covered a broad range of interventions. The large majority of them pertained to the evaluation of small molecules or biologics and most were interventions that had already been tested or approved for use in humans. However, a small number of trials addressed interventions without human precedent of use. Our assessment revealed three major insights about our population of eligible trials: low sample sizes limiting statistical power; increased leniency of response criteria over time; significant underreporting of outcomes and other important study design flaws.

Overall, we observed extremely low sample sizes in most studies, in line with a prior assessment of power and sample size in canine and feline trials^13^. This was true for total study size, size by intervention arm, as well as size broken down by tumor type. We observed that it was common for trials to evaluate the effect of an intervention on multiple tumor types within the same study. However, the low sample size per tumor type per study suggests that these trials may not have had sufficient power to detect differences in effect across multiple tumor types. The typical trial would be able to detect only unrealistically super-effective treatments both for survival and disease progression and few trials had sufficient power to detect very effective treatments. Indeed, based on the median HRs, current clinical trials in dogs were only sufficiently powered to detect a clinical benefit in 1 of the 18 oncology drugs approved by the FDA between 2000 and 2011^35^, if effect sizes in dogs were similar to those seen in humans. Current trials mostly give some preliminary data on how these drugs perform in dogs, but the estimated effect sizes carry very large uncertainty.

This may be acceptable for repurposing human drugs for use in dogs, since they typically already have extensive evidence from their previous use in humans; moreover, having some clinical trial evidence in dogs may be better than having no veterinary evidence at all, at least from a feasibility and safety standpoint. However, the above argument assumes considerable overlap in disease biology and physiology between the two species, which may not necessarily hold true in all cases. Furthermore, if the primary goal is to augment the drug development pipeline (i.e. test new drugs and biologics in veterinary clinical trials before testing them in humans), such very small sample sizes are not very informative. The sample sizes for interventions not previously licensed in humans were not observed to be appreciably larger.

The field would benefit from the conduct of larger trials using consortia and multi-center mechanisms, as has been done in human trials. This approach is also starting to be adopted for murine trials. The recently established Comparative Oncology Trials Consortium (COTC) is an encouraging step in the right direction^2,4,9^. However, all completed trials conducted by the COTC had a target enrollment of between 15–30, with the exception of one trial with a target enrollment of 80–90^9^. This indicates that there may be issues beyond awareness and collaboration that continue to limit sample size even in a multi-center setting, for instance, limited resources or difficulty in recruiting willing owners and eligible participants.

Of note, one might expect that the number of events and hence detectable HR for disease progression would be higher than that of overall survival. However, the results show the opposite. We believe that the observed data arose because there were many more studies reporting disease progression outcomes alone. We observed that a large proportion of the studies that listed only disease progression outcomes were smaller than the ones that listed both progression and survival outcomes. It is these small studies reporting only progression outcomes that led to a lower detectable HR for progression than survival.

We observed that studies which did not adopt the VCOG cRECIST v1.0 guidelines adopted a variety of different thresholds for progressive disease. These thresholds were evenly split between being more and less stringent than VCOG cRECIST. In addition, we identified studies that failed to define the criterion or size metric used to measure disease progression.

In light of these observations, we largely applaud the widespread adoption of standards such as RECIST. Standardization makes comparative assessments across trials more straightforward. Beyond establishing a common set of thresholds, RECIST also defines a standardized methodology for measurement of lesions and tumors. For example, it includes clear guidance on methods that can and cannot be used for lesion measurement^31^. This enables the comparison of multiple studies and interventions, which was previously not possible due to heterogeneity of methods. However, it is crucial to recognize that these response thresholds are arbitrarily set with no inherent biological significance^34^. The level of tolerance for low efficacy may have to shift depending on a multitude of factors. This could include the efficacy of alternative therapies available, as well as the known adverse events associated with the investigational therapy. Candidate drugs with a lower efficacy are more likely to be deemed worthy of further investigation if no effective alternatives are available, or if they present a lower incidence of adverse events compared with the standard of care. Therefore, having continuous, detailed data on tumor size changes alongside RECIST-defined progression and response data would likely improve the ability to assess treatment efficacy with granularity, facilitating comparison with other drugs and potential interventions.

We observed a high degree of unreported experimental details as well as undefined outcomes in the clinical trials in dogs. In comparison, an analysis of 20920 human clinical trials conducted from 1986 to 2014 showed a similar proportion of unreported details for human trials for random sequence generation and allocation concealment; and a lower proportion of unreported details for the remaining categories^36^. The most recent human trials published between 2011 and 2014 showed a lower proportion of underreporting for all categories^36^. Clinical trials in dogs may lag behind human trials in this aspect, however, comparisons need to be tempered by the fact that even for human trials there is large variability in the risk of bias items across different disciplines.

Lack of methodological transparency is a considerable impediment for veterinarians hoping to draw trustworthy conclusions, and for investigators looking to prioritize interventions from veterinary trials into human studies. Moreover, the lack of reported details could be symptomatic of a more pernicious problem. Instead of being due to a simple lack of reporting, this might point to an outright failure to consider these sources of bias, or to define primary outcomes during planning. If these common sources of bias are frequently overlooked in this field, the internal validity of such veterinary clinical trial results might be at high risk. The relationship between lack of reporting and biased estimates of efficacy has been well explored. Studies that do not report experimental details, such as blinding, randomization, and allocation concealment, are consistently observed to yield larger estimates of efficacy. This phenomenon was observed in rodent models^37^, veterinary trials^17^, and human trials^38^. Likewise, the observation that primary outcomes are seldom defined begets questions about the prevalence of post hoc manipulation of outcomes and p-hacking practices^39,40^ in companion animal trials. Beyond the implications for veterinary medicine, such suspicions could stand to prevent such trials from being seriously considered as a trustworthy link in the drug development pathway.

Not reporting methodological details does not necessarily mean that a trial did not use proper methodological safeguards. A study using self-volunteered information from authors found that human trials which failed to report some experimental details actually utilized these approaches in execution^41^. In one evaluation, 96% (52/54) of studies that failed to report concealment of allocation undertook allocation concealment measures in reality^41^. This proportion was 79% (64/81) for blinding of outcome assessors but only 20% for blinding of participants^41^ (5/25). Nonetheless, this finding should not be presumed to hold equally true for studies in dogs. CONSORT reporting guidelines for human randomized controlled trials were first proposed in 1993^42^, the same year in which the Cochrane Collaboration was founded^43^. The field has had two decades to critique issues, design standards, and change mindsets. Though some effort has been made in recent years^13,14,16,19,44^, veterinary trials have not historically received the same scrutiny and emphasis as human trials, and as such there is no guarantee that studies which failed to report details have indeed implemented sufficient bias safeguards. Therefore, even with this qualification, the very presence of high degree of unreported details contributes to uncertainty about the quality of canine trials and should be addressed. Journals and editors could take the lead by requesting adoption of more informative reporting on major design aspects that can improve the quality of trials with translational benefit.

In human trials, reporting standards have increased the proportions of important experimental details in publications^45–47^. Other community-initiated policies, such as the mandatory registration of trial design initiated by the International Committee of Medical Journal Editors (ICMJE)^48^ resulted in rapid increases in registration and improved compliance^49,50^. The aforementioned analysis of 20920 human clinical trials showed a steady reduction in the number of studies at unclear risk over time, across all categories in the Cochrane risk of bias assessment^36^.

Adoption of registration for veterinary clinical trials may also have a similar positive impact in this regard^12^. The American Veterinary Medical Association Animal Health Studies Database (AAHSD), which was launched in 2016, includes 345 entries of trials in dogs as of December 2018. However, unlike the ICMJE policy in human trials, registration is not required for veterinary clinical trials^12^. Furthermore, only 1 registered canine oncology trial had results available with a link to the publication. In addition, the AAHSD is only limited to studies conducted in the United States, Canada, and the United Kingdom^51^, further limiting the adoption of registration in veterinary medicine. Improved transparency may also allow the field to map itself better, enhance visibility of trials and provide stimuli for collaboration towards larger studies in the field.

Additionally, raw, individual patient level data was not always made available in the analyzed studies. None of the five biggest trials reported raw data^52–56^. Smaller trials were more varied, with some reporting individual patient level data^57–59^, while others did not^60,61^. The presence of such raw data might allow pooling of data across studies for analysis, which could be especially relevant in light of the low sample sizes observed.

Experience from human trials shows that often, optional data elements may be missing from registrations, most commonly involving primary and secondary outcomes as well as trial end dates^49,62^. In addition, differences in protocol details can be observed between the registry and published data in a substantial portion of trials^62^. Again, the existing experience from human trials may help to inform efforts in trials in dogs, especially regarding the effectiveness of solutions and ways in which these issues can be pre-emptively adverted.

Some limitations should be discussed. Clinical trials in domestic dogs are not typically reported in phases, unlike human trials^2^. As such, we were unable to distinguish exploratory, early stage trials from late stage trials in our HR analysis. The bulk of small trials with low detectable HRs could well be exploratory trials where statistical power is less critical than late stage trials meant to assess efficacy. Likewise, in the considerable proportion of trials evaluating licensed human therapies for use in dogs, statistical power may be less critical than in trials aiming to translate untested therapies for use in humans. This is because the existing body of efficacy data in humans for repurposed drugs could help address the uncertainty of estimates derived from small veterinary trials. Nonetheless, even with this caveat, the results still hold that the vast majority of trials in dogs have very limited statistical power.

In examining the population of companion dog trials evaluating drugs or biologics not previously approved for humans, we are unable to distinguish trials conducted with the aim of translating therapies for human use from those conducted for the licensing of veterinary-specific treatments. Nonetheless, both types of trials are being conducted by the same community of investigators regardless of primary objective, thus we believe that these insights remain broadly applicable.

Finally, we chose to focus on trials conducted on companion dogs with cancer due to the rapidly increasing prominence of this field. Indeed, all completed and planned trials by the COTC recruited dogs. However, more work should be conducted to determine if our findings are generalizable to other veterinary fields that focus on other animals or other disease types.

It would also be interesting to assess in future work the extent to which results of clinical studies in dogs are predictive of results in human trials. This would require ideally the evaluation of trials that were intended to precede human experimentation versus subsequent human trials. However, even with trials where the companion animal study has typically followed human use, it may be possible to examine whether animal and human trials of the same intervention and for the same cancer yield similar estimates of effect sizes. This will likely require the availability of more, larger trials in dogs with less uncertainty about effect size estimates in order to get more definitive evidence. Beyond prediction of efficacy in humans, other proposed applications of veterinary trials, such as the establishment of biomarkers or testing of drug delivery, should also be assessed empirically in the future, as a larger body of literature on these fronts becomes available. These will be crucial in helping to maximize the translational potential of such trials as part of the drug discovery pipeline.

In closing, our comprehensive assessment of clinical trials in tumor bearing companion dogs reveals numerous areas for improvement. These areas comprise low sample sizes, unclear reporting of response criteria and possible increased leniency over time, a high degree of underreporting of key protocol details outlined in the Cochrane risk of bias tool, and prevalent failure to define primary outcomes. This study builds upon the existing body of evidence examining similar pitfalls in veterinary studies^13,14,16–19^, underscoring the importance of addressing these shortcomings for this research community. In particular, with trials on dogs starting to play a role in human drug discovery, this is an especially opportune time for increased awareness and improvements to the conduct of research.

## Supplementary information

Supplementary Appendix 1

## Data Availability

Data and analysis scripts used in this study have been deposited on the Open Science Framework and are accessible at https://osf.io/5e37z/?view_only=e70e94fbe6824c9cac75c3e1e03a1c5b.
