# Supplementary material for: An empirical assessment of research practices across 163 clinical trials of tumor-bearing companion dogs
Source: Sci Rep. 2019 Aug 15;9:11877. doi: 10.1038/s41598-019-48425-5 (PMC6695388; doi:10.1038/s41598-019-48425-5)
Supplement: Supplementary file 1 — Supplementary Appendix 1 [file 41598_2019_48425_MOESM1_ESM.pdf]

An empirical assessment of research practices across 163 clinical trials of tumor-bearing companion dogs

Supplementary material

Yuan Jin Tan, BS[1,4], Ryan J. Crowley[2,4], John P.A. Ioannidis, MD, DSc [1,2,3,4] *

1. Department of Health Research and Policy, Division of Epidemiology, Stanford School of Medicine, Stanford, CA, USA
2. Department of Statistics, Stanford University School of Humanities and Sciences, Stanford, CA, USA
3. Stanford Prevention Research Center, Department of Medicine, and Department of Biomedical Data Science, Stanford University School of Medicine, Stanford, CA, USA
4. Meta-Research Innovation Center at Stanford (METRICS), Stanford University, Stanford, CA, USA

* Address correspondence to: John P.A. Ioannidis, MD, DSc, Stanford Prevention Research Center and Meta-Research Innovation Center at Stanford (METRICS), 1265 Welch Rd, Medical School Office Building, Room X306, Stanford CA 94305, USA. E-mail: jioannid@stanford.edu

Supplementary Appendix 1

References list of included trials

1. Bergman, P. J. *et al.* Development of a xenogeneic DNA vaccine program for canine malignant melanoma at the Animal Medical Center. *Vaccine* **24**, 4582–4585 (2006).
2. Meyer, J. A. *et al.* Canine osteogenic sarcoma treated by amputation and MER: an adverse effect of splenectomy on survival. *Cancer* **49**, 1613–1616 (1982).
3. Dewhirst, M. W., Connor, W. G. & Sim, D. A. Preliminary results of a phase III trial of spontaneous animal tumors to heat and/or radiation: early normal tissue response and tumor volume influence on initial response. *Int. J. Radiat. Oncol. Biol. Phys.* **8**, 1951–1961 (1982).
4. Dewhirst, M. W. & Sim, D. A. The utility of thermal dose as a predictor of tumor and normal tissue responses to combined radiation and hyperthermia. *Cancer Res.* **44**, 4772s–4780s (1984).
5. Dewhirst, M. W. *et al.* Local control and distant metastases in primary canine malignant melanomas treated with hyperthermia and/or radiotherapy. *Int J Hyperthermia* **1**, 219–234 (1985).
6. MacEwen, E. G. *et al.* Levamisole as adjuvant to chemotherapy for canine lymphosarcoma. *J Biol Response Mod* **4**, 427–433 (1985).
7. MacEwen, E. G. *et al.* Evaluation of effects of levamisole and surgery on canine mammary cancer. *J Biol Response Mod* **4**, 418–426 (1985).
8. McChesney, S. L., Gillette, E. L., Dewhirst, M. W. & Withrow, S. J. Influence of WR 2721 on radiation response of canine soft tissue sarcomas. *Int. J. Radiat. Oncol. Biol. Phys.* **12**, 1957–1963 (1986).
9. Misdorp, W. Incomplete surgery, local immunostimulation, and recurrence of some tumour types in dogs and cats. *Vet Q* **9**, 279–286 (1987).
10. Gillette, E. L., McChesney, S. L., Dewhirst, M. W. & Scott, R. J. Response of canine oral carcinomas

to heat and radiation. *Int. J. Radiat. Oncol. Biol. Phys.* **13**, 1861–1867 (1987).

11. MacEwen, E. G. *et al.* Therapy for osteosarcoma in dogs with intravenous injection of liposome-encapsulated muramyl tripeptide. *J. Natl. Cancer Inst.* **81**, 935–938 (1989).

12. Teske, E., Rutteman, G. R., van Heerde, P. & Misdorp, W. Polyethylene glycol-L-asparaginase versus native L-asparaginase in canine non-Hodgkin's lymphoma. *Eur. J. Cancer* **26**, 891–895 (1990).

13. Brezovich, I. A. *et al.* Hyperthermia of pet animal tumours with self-regulating ferromagnetic thermoseeds. *Int J Hyperthermia* **6**, 117–130 (1990).

14. Grier, R. L. *et al.* Mast cell tumor destruction by deionized water. *Am. J. Vet. Res.* **51**, 1116–1120 (1990).

15. Page, R. L. *et al.* Phase I study of melphalan alone and melphalan plus whole body hyperthermia in dogs with malignant melanoma. *Int J Hyperthermia* **7**, 559–566 (1991).

16. Denman, D. L. *et al.* Therapeutic responses of spontaneous canine malignancies to combinations of radiotherapy and hyperthermia. *Int. J. Radiat. Oncol. Biol. Phys.* **21**, 415–422 (1991).

17. Knapp, D. W., Richardson, R. C., Bottoms, G. D., Teclaw, R. & Chan, T. C. Phase I trial of piroxicam in 62 dogs bearing naturally occurring tumors. *Cancer Chemother. Pharmacol.* **29**, 214–218 (1992).

18. Page, R. L. *et al.* Phase III evaluation of doxorubicin and whole-body hyperthermia in dogs with lymphoma. *Int J Hyperthermia* **8**, 187–197 (1992).

19. Gillette, S. M. *et al.* Response of canine soft tissue sarcomas to radiation or radiation plus hyperthermia: a randomized phase II study. *Int J Hyperthermia* **8**, 309–320 (1992).

20. MacEwen, E. G., Rosenthal, R. C., Fox, L. E., Loar, A. S. & Kurzman, I. D. Evaluation of L-asparaginase: polyethylene glycol conjugate versus native L-asparaginase combined with

chemotherapy. A randomized double-blind study in canine lymphoma. *J. Vet. Intern. Med.* **6**, 230–234 (1992).

21. Novotney, C. A. *et al.* Phase I evaluation of doxorubicin and whole-body hyperthermia in dogs with lymphoma. *J. Vet. Intern. Med.* **6**, 245–249 (1992).

22. Page, R. L. *et al.* Quantitative estimation of the thermal dose-modifying factor for cis-diamminedichloroplatinum (CDDP) in tumour-bearing dogs. *Int J Hyperthermia* **8**, 761–769 (1992).

23. Ogilvie, G. K. *et al.* Energy expenditure in dogs with lymphoma fed two specialized diets. *Cancer* **71**, 3146–3152 (1993).

24. Prescott, D. M. *et al.* Manipulation of intra- and extracellular pH in spontaneous canine tumours by use of hyperglycaemia. *Int J Hyperthermia* **9**, 745–754 (1993).

25. Moore, A. S., Rand, W. M., Berg, J., L'Heureux, D. A. & Dennis, R. A. Evaluation of butorphanol and cyproheptadine for prevention of cisplatin-induced vomiting in dogs. *J. Am. Vet. Med. Assoc.* **205**, 441–443 (1994).

26. Page, R. L. *et al.* Effect of whole body hyperthermia on carboplatin disposition and toxicity in dogs. *Int J Hyperthermia* **10**, 807–816 (1994).

27. Frew, D. G., Dobson, J. M., Stenning, S. P. & Bleehen, N. M. Response of 145 spontaneous canine head and neck tumours to radiation versus radiation plus microwave hyperthermia: results of a randomized phase III clinical study. *Int J Hyperthermia* **11**, 217–230 (1995).

28. Vail, D. M. *et al.* Liposome-encapsulated muramyl tripeptide phosphatidylethanolamine adjuvant immunotherapy for splenic hemangiosarcoma in the dog: a randomized multi-institutional clinical trial. *Clin. Cancer Res.* **1**, 1165–1170 (1995).

29. Kurzman, I. D. *et al.* Adjuvant therapy for osteosarcoma in dogs: results of randomized clinical

trials using combined liposome-encapsulated muramyl tripeptide and cisplatin. *Clin. Cancer Res.* **1**, 1595–1601 (1995).

30. Thrall, D. E. *et al.* Radiation plus local hyperthermia versus radiation plus the combination of local and whole-body hyperthermia in canine sarcomas. *Int. J. Radiat. Oncol. Biol. Phys.* **34**, 1087–1096 (1996).
31. Hauck, M. L. *et al.* Phase I evaluation of mitoxantrone alone and combined with whole body hyperthermia in dogs with lymphoma. *Int J Hyperthermia* **12**, 309–320 (1996).
32. Hahn, K. A., Legendre, A. M. & Schuller, H. M. Amputation and dexniguldipine as treatment for canine appendicular osteosarcoma. *J. Cancer Res. Clin. Oncol.* **123**, 34–38 (1997).
33. Berg, J., Gebhardt, M. C. & Rand, W. M. Effect of timing of postoperative chemotherapy on survival of dogs with osteosarcoma. *Cancer* **79**, 1343–1350 (1997).
34. Teske, E., Rutteman, G. R., vd Ingh, T. S., van Noort, R. & Misdorp, W. Liposome-encapsulated muramyl tripeptide phosphatidylethanolamine (L-MTP-PE): a randomized clinical trial in dogs with mammary carcinoma. *Anticancer Res.* **18**, 1015–1019 (1998).
35. Vail, D. M. *et al.* Efficacy of pyridoxine to ameliorate the cutaneous toxicity associated with doxorubicin containing pegylated (Stealth) liposomes: a randomized, double-blind clinical trial using a canine model. *Clin. Cancer Res.* **4**, 1567–1571 (1998).
36. Khanna, C. *et al.* Randomized controlled trial of doxorubicin versus dactinomycin in a multiagent protocol for treatment of dogs with malignant lymphoma. *J. Am. Vet. Med. Assoc.* **213**, 985–990 (1998).
37. Valerius, K. D. *et al.* Comparison of the effects of asparaginase administered subcutaneously versus intramuscularly for treatment of multicentric lymphoma in dogs receiving doxorubicin. *J. Am. Vet. Med. Assoc.* **214**, 353–356 (1999).

38. Lombardi, P., Florio, S., Pagnini, U., Crispino, A. & Avallone, L. Ovarian function suppression with a GnRH analogue: D-ser(But[t])[6]-Arzgly[10]-LHRH (Goserelin) in hormone dependent canine mammary cancer. *J. Vet. Pharmacol. Ther.* **22**, 56–61 (1999).

39. Thrall, D. E. *et al.* Use of whole body hyperthermia as a method to heat inaccessible tumours uniformly: a phase III trial in canine brain masses. *Int J Hyperthermia* **15**, 383–398 (1999).

40. McNiel, E. A., Ogilvie, G. K., Mallinckrodt, C., Richardson, K. & Fettman, M. J. Platelet function in dogs treated for lymphoma and hemangiosarcoma and supplemented with dietary n-3 fatty acids. *J. Vet. Intern. Med.* **13**, 574–580 (1999).

41. Larue, S. M. *et al.* Tumour cell kinetics as predictors of response in canine lymphoma treated with chemotherapy alone or combined with whole body hyperthermia. *Int J Hyperthermia* **15**, 475–486 (1999).

42. MacEwen, E. G. *et al.* Adjuvant therapy for melanoma in dogs: results of randomized clinical trials using surgery, liposome-encapsulated muramyl tripeptide, and granulocyte macrophage colony-stimulating factor. *Clin. Cancer Res.* **5**, 4249–4258 (1999).

43. Ogilvie, G. K. *et al.* Effect of fish oil, arginine, and doxorubicin chemotherapy on remission and survival time for dogs with lymphoma: a double-blind, randomized placebo-controlled study. *Cancer* **88**, 1916–1928 (2000).

44. Thrall, D. E. *et al.* Using units of CEM 43 degrees C T90, local hyperthermia thermal dose can be delivered as prescribed. *Int J Hyperthermia* **16**, 415–428 (2000).

45. Knapp, D. W. *et al.* Cisplatin versus cisplatin combined with piroxicam in a canine model of human invasive urinary bladder cancer. *Cancer Chemother. Pharmacol.* **46**, 221–226 (2000).

46. Khanna, C. *et al.* A randomized controlled trial of octreotide pamoate long-acting release and carboplatin versus carboplatin alone in dogs with naturally occurring osteosarcoma: evaluation

of insulin-like growth factor suppression and chemotherapy. *Clin. Cancer Res.* **8**, 2406–2412 (2002).

47. Vail, D. M. *et al.* STEALTH liposome-encapsulated cisplatin (SPI-77) versus carboplatin as adjuvant therapy for spontaneously arising osteosarcoma (OSA) in the dog: a randomized multicenter clinical trial. *Cancer Chemother. Pharmacol.* **50**, 131–136 (2002).
48. Draghia-Akli, R., Hahn, K. A., King, G. K., Cummings, K. K. & Carpenter, R. H. Effects of plasmid-mediated growth hormone-releasing hormone in severely debilitated dogs with cancer. *Mol. Ther.* **6**, 830–836 (2002).
49. Bergman, P. J. *et al.* Long-term survival of dogs with advanced malignant melanoma after DNA vaccination with xenogeneic human tyrosinase: a phase I trial. *Clin. Cancer Res.* **9**, 1284–1290 (2003).
50. Withrow, S. J. *et al.* Biodegradable cisplatin polymer in limb-sparing surgery for canine osteosarcoma. *Ann. Surg. Oncol.* **11**, 705–713 (2004).
51. Poulson, J. M. *et al.* Effect of calcitonin gene related peptide vs sodium nitroprusside to increase temperature in spontaneous canine tumours during local hyperthermia. *Int J Hyperthermia* **20**, 477–489 (2004).
52. Henry, C. J. *et al.* Clinical evaluation of BR96 sFv-PE40 immunotoxin therapy in canine models of spontaneously occurring invasive carcinoma. *Clin. Cancer Res.* **11**, 751–755 (2005).
53. Thrall, D. E. *et al.* Thermal dose is related to duration of local control in canine sarcomas treated with thermoradiotherapy. *Clin. Cancer Res.* **11**, 5206–5214 (2005).
54. Kamstock, D. *et al.* Liposome-DNA complexes infused intravenously inhibit tumor angiogenesis and elicit antitumor activity in dogs with soft tissue sarcoma. *Cancer Gene Ther.* **13**, 306–317 (2006).

55. Frimberger, A. E. *et al.* A combination chemotherapy protocol with dose intensification and autologous bone marrow transplant (VELCAP-HDC) for canine lymphoma. *J. Vet. Intern. Med.* **20**, 355–364 (2006).

56. Steagall, P. V. M., Teixeira Neto, F. J., Minto, B. W., Campagnol, D. & Corrêa, M. A. Evaluation of the isoflurane-sparing effects of lidocaine and fentanyl during surgery in dogs. *J. Am. Vet. Med. Assoc.* **229**, 522–527 (2006).

57. Liptak, J. M. *et al.* Cortical allograft and endoprosthesis for limb-sparing surgery in dogs with distal radial osteosarcoma: a prospective clinical comparison of two different limb-sparing techniques. *Vet Surg* **35**, 518–533 (2006).

58. Vlasin, M., Rauser, P., Fichtel, T. & Necas, A. Subtotal intracapsular prostatectomy as a useful treatment for advanced-stage prostatic malignancies. *J Small Anim Pract* **47**, 512–516 (2006).

59. Simon, D., Schoenrock, D., Baumgärtner, W. & Nolte, I. Postoperative adjuvant treatment of invasive malignant mammary gland tumors in dogs with doxorubicin and docetaxel. *J. Vet. Intern. Med.* **20**, 1184–1190 (2006).

60. Rusk, A. *et al.* Cooperative activity of cytotoxic chemotherapy with antiangiogenic thrombospondin-I peptides, ABT-526 in pet dogs with relapsed lymphoma. *Clin. Cancer Res.* **12**, 7456–7464 (2006).

61. Vail, D. M., Rodabaugh, H. S., Conder, G. A., Boucher, J. F. & Mathur, S. Efficacy of injectable maropitant (Cerenia) in a randomized clinical trial for prevention and treatment of cisplatin-induced emesis in dogs presented as veterinary patients. *Vet Comp Oncol* **5**, 38–46 (2007).

62. Chretin, J. D. *et al.* Prophylactic trimethoprim-sulfadiazine during chemotherapy in dogs with lymphoma and osteosarcoma: a double-blind, placebo-controlled study. *J. Vet. Intern. Med.* **21**, 141–148 (2007).

63. Borgatti-Jeffreys, A., Hooser, S. B., Miller, M. A. & Lucroy, M. D. Phase I clinical trial of the use of zinc phthalocyanine tetrasulfonate as a photosensitizer for photodynamic therapy in dogs. *Am. J. Vet. Res.* **68**, 399–404 (2007).

64. DiResta, G. R. *et al.* Use of an artificial lymphatic system during carboplatin infusion to improve canine osteosarcoma blood flow and clinical response. *Ann. Surg. Oncol.* **14**, 2411–2421 (2007).

65. Flynn, A. K., Lurie, D. M., Ward, J., Lewis, D. T. & Marsella, R. The clinical and histopathological effects of prednisone on acute radiation-induced dermatitis in dogs: a placebo-controlled, randomized, double-blind, prospective clinical trial. *Vet. Dermatol.* **18**, 217–226 (2007).

66. Dervisis, N. G. *et al.* Efficacy of temozolomide or dacarbazine in combination with an anthracycline for rescue chemotherapy in dogs with lymphoma. *J. Am. Vet. Med. Assoc.* **231**, 563–569 (2007).

67. Moore, A. S. *et al.* Doxorubicin and BAY 12-9566 for the treatment of osteosarcoma in dogs: a randomized, double-blind, placebo-controlled study. *J. Vet. Intern. Med.* **21**, 783–790 (2007).

68. Hermo, G. A. *et al.* Perioperative desmopressin prolongs survival in surgically treated bitches with mammary gland tumours: a pilot study. *Vet. J.* **178**, 103–108 (2008).

69. Greene, S. N., Lucroy, M. D., Greenberg, C. B., Bonney, P. L. & Knapp, D. W. Evaluation of cisplatin administered with piroxicam in dogs with transitional cell carcinoma of the urinary bladder. *J. Am. Vet. Med. Assoc.* **231**, 1056–1060 (2007).

70. Henry, C. J. *et al.* Evaluation of a novel immunomodulator composed of human chorionic gonadotropin and bacillus Calmette-Guerin for treatment of canine mast cell tumors in clinically affected dogs. *Am. J. Vet. Res.* **68**, 1246–1251 (2007).

71. Telló, M. *et al.* Electrochemical therapy to treat cancer (in vivo treatment). *Conf Proc IEEE Eng Med Biol Soc* **2007**, 3524–3527 (2007).

72. Turek, M. M. *et al.* Human granulocyte-macrophage colony-stimulating factor DNA cationic-lipid complexed autologous tumour cell vaccination in the treatment of canine B-cell multicentric lymphoma. *Vet Comp Oncol* **5**, 219–231 (2007).

73. Rassnick, K. M. *et al.* Comparison of 3 protocols for treatment after induction of remission in dogs with lymphoma. *J. Vet. Intern. Med.* **21**, 1364–1373 (2007).

74. Simon, D. *et al.* Efficacy of a continuous, multiagent chemotherapeutic protocol versus a short-term single-agent protocol in dogs with lymphoma. *J. Am. Vet. Med. Assoc.* **232**, 879–885 (2008).

75. Bodles-Brakhop, A. M., Brown, P. A., Pope, M. A. & Draghia-Akli, R. Double-blinded, Placebo-controlled plasmid GHRH trial for cancer-associated anemia in dogs. *Mol. Ther.* **16**, 862–870 (2008).

76. Hahn, K. A. *et al.* Masitinib is safe and effective for the treatment of canine mast cell tumors. *J. Vet. Intern. Med.* **22**, 1301–1309 (2008).

77. Brocks, B. A. W., Neyens, I. J. S., Teske, E. & Kirpensteijn, J. Hypotonic water as adjuvant therapy for incompletely resected canine mast cell tumors: a randomized, double-blind, placebo-controlled study. *Vet Surg* **37**, 472–478 (2008).

78. Rassnick, K. M. *et al.* Efficacy of vinblastine for treatment of canine mast cell tumors. *J. Vet. Intern. Med.* **22**, 1390–1396 (2008).

79. Fan, T. M. *et al.* Double-blind placebo-controlled trial of adjuvant pamidronate with palliative radiotherapy and intravenous doxorubicin for canine appendicular osteosarcoma bone pain. *J. Vet. Intern. Med.* **23**, 152–160 (2009).

80. Marconato, L., Lorenzo, R. M., Abramo, F., Ratto, A. & Zini, E. Adjuvant gemcitabine after surgical removal of aggressive malignant mammary tumours in dogs. *Vet Comp Oncol* **6**, 90–101 (2008).

81. Lurie, D. M., Kent, M. S., Fry, M. M. & Théon, A. P. A toxicity study of low-dose rate half-body

irradiation and chemotherapy in dogs with lymphoma. *Vet Comp Oncol* **6**, 257–267 (2008).

82. Vail, D. M. *et al.* Assessment of GS-9219 in a pet dog model of non-Hodgkin's lymphoma. *Clin. Cancer Res.* **15**, 3503–3510 (2009).

83. Wright, Z. *et al.* A pilot study evaluating changes in pancreatic lipase immunoreactivity concentrations in canines treated with L-asparaginase (ASNase), vincristine, or both for lymphoma. *Can. J. Vet. Res.* **73**, 103–110 (2009).

84. London, C. A. *et al.* Multi-center, placebo-controlled, double-blind, randomized study of oral toceranib phosphate (SU11654), a receptor tyrosine kinase inhibitor, for the treatment of dogs with recurrent (either local or distant) mast cell tumor following surgical excision. *Clin. Cancer Res.* **15**, 3856–3865 (2009).

85. Lawrence, J. *et al.* Use of 3'-deoxy-3'-[18F]fluorothymidine PET/CT for evaluating response to cytotoxic chemotherapy in dogs with non-Hodgkin's lymphoma. *Vet Radiol Ultrasound* **50**, 660–668 (2009).

86. Lori, J. C., Stein, T. J. & Thamm, D. H. Doxorubicin and cyclophosphamide for the treatment of canine lymphoma: a randomized, placebo-controlled study. *Vet Comp Oncol* **8**, 188–195 (2010).

87. Abbo, A. H. *et al.* Phase I clinical trial and pharmacokinetics of intravesical mitomycin C in dogs with localized transitional cell carcinoma of the urinary bladder. *J. Vet. Intern. Med.* **24**, 1124–1130 (2010).

88. Rau, S. E., Barber, L. G. & Burgess, K. E. Efficacy of maropitant in the prevention of delayed vomiting associated with administration of doxorubicin to dogs. *J. Vet. Intern. Med.* **24**, 1452–1457 (2010).

89. Hermo, G. A. *et al.* Effect of adjuvant perioperative desmopressin in locally advanced canine mammary carcinoma and its relation to histologic grade. *J Am Anim Hosp Assoc* **47**, 21–27

(2011).

90. Blankenbecler, R. Low-dose pretreatment for radiation therapy. *Dose Response* **8**, 534–542 (2010).

91. Guil-Luna, S. *et al.* Aglepristone decreases proliferation in progesterone receptor-positive canine mammary carcinomas. *J. Vet. Intern. Med.* **25**, 518–523 (2011).

92. Warry, E., Hansen, R. J., Gustafson, D. L. & Lana, S. E. Pharmacokinetics of cyclophosphamide after oral and intravenous administration to dogs with lymphoma. *J. Vet. Intern. Med.* **25**, 903–908 (2011).

93. Henson, M. S. *et al.* Immunotherapy with autologous tumour antigen-coated microbeads (large multivalent immunogen), IL-2 and GM-CSF in dogs with spontaneous B-cell lymphoma. *Vet Comp Oncol* **9**, 95–105 (2011).

94. Skorupski, K. A. *et al.* Prospective randomized clinical trial assessing the efficacy of Denamarin for prevention of CCNU-induced hepatopathy in tumor-bearing dogs. *J. Vet. Intern. Med.* **25**, 838–845 (2011).

95. London, C. A. *et al.* Phase I evaluation of STA-1474, a prodrug of the novel HSP90 inhibitor ganetespib, in dogs with spontaneous cancer. *PLoS ONE* **6**, e27018 (2011).

96. Teske, E., Rutteman, G. R., Kirpenstein, J. & Hirschberger, J. A randomized controlled study into the efficacy and toxicity of pegylated liposome encapsulated doxorubicin as an adjuvant therapy in dogs with splenic haemangiosarcoma. *Vet Comp Oncol* **9**, 283–289 (2011).

97. Hahn, N. M. *et al.* Subcutaneous 5-azacitidine treatment of naturally occurring canine urothelial carcinoma: a novel epigenetic approach to human urothelial carcinoma drug development. *J. Urol.* **187**, 302–309 (2012).

98. Hansen, R. A. *et al.* Menhaden oil administration to dogs treated with radiation for nasal tumors

demonstrates lower levels of tissue eicosanoids. *Nutr Res* **31**, 929–936 (2011).

99. Krick, E. L. *et al.* Evaluation of Clostridium novyi-NT spores in dogs with naturally occurring tumors. *Am. J. Vet. Res.* **73**, 112–118 (2012).

100. Spugnini, E. P. *et al.* Lansoprazole as a rescue agent in chemoresistant tumors: a phase I/II study in companion animals with spontaneously occurring tumors. *J Transl Med* **9**, 221 (2011).

101. Keyerleber, M. A., Gieger, T. L., Erb, H. N., Thompson, M. S. & McEntee, M. C. Three-dimensional conformal versus non-graphic radiation treatment planning for apocrine gland adenocarcinoma of the anal sac in 18 dogs (2002-2007). *Vet Comp Oncol* **10**, 237–245 (2012).

102. Robat, C. *et al.* Safety evaluation of combination vinblastine and toceranib phosphate (Palladia®) in dogs: a phase I dose-finding study. *Vet Comp Oncol* **10**, 174–183 (2012).

103. Chon, E., McCartan, L., Kubicek, L. N. & Vail, D. M. Safety evaluation of combination toceranib phosphate (Palladia®) and piroxicam in tumour-bearing dogs (excluding mast cell tumours): a phase I dose-finding study. *Vet Comp Oncol* **10**, 184–193 (2012).

104. Vail, D. M. *et al.* A randomized trial investigating the efficacy and safety of water soluble micellar paclitaxel (Paccal Vet) for treatment of nonresectable grade 2 or 3 mast cell tumors in dogs. *J. Vet. Intern. Med.* **26**, 598–607 (2012).

105. Vail, D. M. *et al.* Phase I study to determine the maximal tolerated dose and dose-limiting toxicities of orally administered idarubicin in dogs with lymphoma. *J. Vet. Intern. Med.* **26**, 608–613 (2012).

106. Michou, J. N., Leece, E. A. & Brearley, J. C. Comparison of pain on injection during induction of anaesthesia with alfaxalone and two formulations of propofol in dogs. *Vet Anaesth Analg* **39**, 275–281 (2012).

107. Lavalle, G. E., De Campos, C. B., Bertagnolli, A. C. & Cassali, G. D. Canine malignant mammary

gland neoplasms with advanced clinical staging treated with carboplatin and cyclooxygenase inhibitors. *In Vivo* **26**, 375–379 (2012).

108. Thrall, D. E. *et al.* Thermal dose fractionation affects tumour physiological response. *Int J Hyperthermia* **28**, 431–440 (2012).

109. Sahora, A. I. *et al.* Prospective study of thrombospondin-1 mimetic peptides, ABT-510 and ABT-898, in dogs with soft tissue sarcoma. *J. Vet. Intern. Med.* **26**, 1169–1176 (2012).

110. Brown, D. C. & Reetz, J. Single agent polysaccharopeptide delays metastases and improves survival in naturally occurring hemangiosarcoma. *Evid Based Complement Alternat Med* **2012**, 384301 (2012).

111. Finocchiaro, L. M. E. & Glikin, G. C. Cytokine-enhanced vaccine and suicide gene therapy as surgery adjuvant treatments for spontaneous canine melanoma: 9 years of follow-up. *Cancer Gene Ther.* **19**, 852–861 (2012).

112. Knapp, D. W. *et al.* Randomized trial of cisplatin versus firocoxib versus cisplatin/firocoxib in dogs with transitional cell carcinoma of the urinary bladder. *J. Vet. Intern. Med.* **27**, 126–133 (2013).

113. Romkes, G., Klopfleisch, R. & Eule, J. C. Evaluation of one- vs. two-layered closure after wedge excision of 43 eyelid tumors in dogs. *Vet Ophthalmol* **17**, 32–40 (2014).

114. Flôr, P. B., Yazbek, K. V. B., Ida, K. K. & Fantoni, D. T. Tramadol plus metamizole combined or not with anti-inflammatory drugs is clinically effective for moderate to severe chronic pain treatment in cancer patients. *Vet Anaesth Analg* **40**, 316–327 (2013).

115. Lucas, S. R. R. *et al.* Pilot clinical study of carmustine associated with a lipid nanoemulsion in combination with vincristine and prednisone for the treatment of canine lymphoma. *Vet Comp Oncol* **13**, 184–193 (2015).

116. Kozicki, A. R., Robat, C., Chun, R. & Kurzman, I. D. Adjuvant therapy with carboplatin and pamidronate for canine appendicular osteosarcoma. *Vet Comp Oncol* **13**, 229–236 (2015).

117. Zandvliet, M., Rutteman, G. R. & Teske, E. Prednisolone inclusion in a first-line multidrug cytostatic protocol for the treatment of canine lymphoma does not affect therapy results. *Vet. J.* **197**, 656–661 (2013).

118. Gavazza, A. *et al.* Safety and efficacy of a genetic vaccine targeting telomerase plus chemotherapy for the therapy of canine B-cell lymphoma. *Hum. Gene Ther.* **24**, 728–738 (2013).

119. Teixeira, R. C. *et al.* Effects of tramadol alone, in combination with meloxicam or dipyrone, on postoperative pain and the analgesic requirement in dogs undergoing unilateral mastectomy with or without ovariohysterectomy. *Vet Anaesth Analg* **40**, 641–649 (2013).

120. Haagsman, A. N., Witkamp, A. C. S., Sjollema, B. E., Kik, M. J. L. & Kirpensteijn, J. The effect of interleukin-2 on canine peripheral nerve sheath tumours after marginal surgical excision: a double-blind randomized study. *BMC Vet. Res.* **9**, 155 (2013).

121. Minto, B. W., Rodrigues, L. C., Steagall, P. V. M., Monteiro, E. R. & Brandão, C. V. S. Assessment of postoperative pain after unilateral mastectomy using two different surgical techniques in dogs. *Acta Vet. Scand.* **55**, 60 (2013).

122. Credie, L. de F. G. A. *et al.* Perioperative evaluation of tumescent anaesthesia technique in bitches submitted to unilateral mastectomy. *BMC Vet. Res.* **9**, 178 (2013).

123. Skorupski, K. A. *et al.* Carboplatin versus alternating carboplatin and doxorubicin for the adjuvant treatment of canine appendicular osteosarcoma: a randomized, phase III trial. *Vet Comp Oncol* **14**, 81–87 (2016).

124. Brown, D. C. & Agnello, K. Intrathecal substance P-saporin in the dog: efficacy in bone cancer pain. *Anesthesiology* **119**, 1178–1185 (2013).

125. Marconato, L. *et al.* Randomized, placebo-controlled, double-blinded chemoimmunotherapy clinical trial in a pet dog model of diffuse large B-cell lymphoma. *Clin. Cancer Res.* **20**, 668–677 (2014).

126. Pan, X., Tsimbas, K., Kurzman, I. D. & Vail, D. M. Safety evaluation of combination CCNU and continuous toceranib phosphate (Palladia(®)) in tumour-bearing dogs: a phase I dose-finding study. *Vet Comp Oncol* **14**, 202–209 (2016).

127. Habineza Ndikuyeze, G. *et al.* A phase I clinical trial of systemically delivered NEMO binding domain peptide in dogs with spontaneous activated B-cell like diffuse large B-cell lymphoma. *PLoS ONE* **9**, e95404 (2014).

128. Withers, S. S. *et al.* Fasting Reduces the Incidence of Delayed-Type Vomiting Associated with Doxorubicin Treatment in Dogs with Lymphoma. *Transl Oncol* (2014). doi:10.1016/j.tranon.2014.04.014

129. Mason, S. L., Grant, I. A., Elliott, J., Cripps, P. & Blackwood, L. Gastrointestinal toxicity after vincristine or cyclophosphamide administered with or without maropitant in dogs: a prospective randomised controlled study. *J Small Anim Pract* **55**, 391–398 (2014).

130. Michałowska, M. *et al.* Phase I/II clinical trial of encapsulated, cytochrome P450 expressing cells as local activators of cyclophosphamide to treat spontaneous canine tumours. *PLoS ONE* **9**, e102061 (2014).

131. Hosoya, K. *et al.* Comparison of high-dose intermittent and low-dose continuous oral artemisinin in dogs with naturally occurring tumors. *J Am Anim Hosp Assoc* **50**, 390–395 (2014).

132. Morges, M. A. *et al.* Phase II evaluation of VDC-1101 in canine cutaneous T-cell lymphoma. *J. Vet. Intern. Med.* **28**, 1569–1574 (2014).

133. Axiak-Bechtel, S. M. *et al.* Gum arabic-coated radioactive gold nanoparticles cause no short-term

local or systemic toxicity in the clinically relevant canine model of prostate cancer. *Int J Nanomedicine* **9**, 5001–5011 (2014).

134. Cancedda, S. *et al.* Efficacy and side effects of radiation therapy in comparison with radiation therapy and temozolomide in the treatment of measurable canine malignant melanoma. *Vet Comp Oncol* **14**, e146–e157 (2016).

135. Allstadt, S. D., Rodriguez, C. O., Boostrom, B., Rebhun, R. B. & Skorupski, K. A. Randomized phase III trial of piroxicam in combination with mitoxantrone or carboplatin for first-line treatment of urogenital tract transitional cell carcinoma in dogs. *J. Vet. Intern. Med.* **29**, 261–267 (2015).

136. Brown, D. C., Agnello, K. & Iadarola, M. J. Intrathecal resiniferatoxin in a dog model: efficacy in bone cancer pain. *Pain* **156**, 1018–1024 (2015).

137. Athanasiadi, I., Geigy, C., Hilger, R. A., Meier, V. & Rohrer Bley, C. Safety, tolerability and pharmacokinetic properties of the novel triazene TriN 2755 in tumour bearing dogs - a phase I study†. *Vet Comp Oncol* **15**, 94–104 (2017).

138. Cancedda, S. *et al.* Combination of radiation therapy and firocoxib for the treatment of canine nasal carcinoma. *Vet Radiol Ultrasound* **56**, 335–343 (2015).

139. Finocchiaro, L. M. E. *et al.* Cytokine-Enhanced Vaccine and Interferon-β plus Suicide Gene Therapy as Surgery Adjuvant Treatments for Spontaneous Canine Melanoma. *Hum. Gene Ther.* **26**, 367–376 (2015).

140. Crociolli, G. C. *et al.* Gabapentin as an adjuvant for postoperative pain management in dogs undergoing mastectomy. *J. Vet. Med. Sci.* **77**, 1011–1015 (2015).

141. Marconato, L. *et al.* An open-label phase 1 dose-escalation clinical trial of a single intravenous administration of gemcitabine in dogs with advanced solid tumors. *J. Vet. Intern. Med.* **29**, 620–625 (2015).

142. London, C. A. *et al.* Impact of Toceranib/Piroxicam/Cyclophosphamide Maintenance Therapy on Outcome of Dogs with Appendicular Osteosarcoma following Amputation and Carboplatin Chemotherapy: A Multi-Institutional Study. *PLoS ONE* **10**, e0124889 (2015).

143. Paoloni, M. *et al.* Defining the Pharmacodynamic Profile and Therapeutic Index of NHS-IL12 Immunocytokine in Dogs with Malignant Melanoma. *PLoS ONE* **10**, e0129954 (2015).

144. Tierny, D. *et al.* Phase I Clinical Pharmacology Study of F14512, a New Polyamine-Vectorized Anticancer Drug, in Naturally Occurring Canine Lymphoma. *Clin. Cancer Res.* **21**, 5314–5323 (2015).

145. Karrasch, N. M., Lerche, P., Aarnes, T. K., Gardner, H. L. & London, C. A. The effects of preoperative oral administration of carprofen or tramadol on postoperative analgesia in dogs undergoing cutaneous tumor removal. *Can. Vet. J.* **56**, 817–822 (2015).

146. Pavelski, M. *et al.* Infrared Thermography in Dogs with Mammary Tumors and Healthy Dogs. *J. Vet. Intern. Med.* **29**, 1578–1583 (2015).

147. Marconato, L. *et al.* Enhanced therapeutic effect of APAVAC immunotherapy in combination with dose-intense chemotherapy in dogs with advanced indolent B-cell lymphoma. *Vaccine* **33**, 5080–5086 (2015).

148. Vilhegas, S. *et al.* Botulinum toxin type A as an adjunct in postoperative pain management in dogs undergoing radical mastectomy. *Vet. Rec.* **177**, 391 (2015).

149. Rasmussen, R. M., Kurzman, I. D., Biller, B. J., Guth, A. & Vail, D. M. Phase I lead-in and subsequent randomized trial assessing safety and modulation of regulatory T cell numbers following a maximally tolerated dose doxorubicin and metronomic dose cyclophosphamide combination chemotherapy protocol in tumour-bearing dogs. *Vet Comp Oncol* **15**, 421–430 (2017).

150. Kristiansen, V. M. *et al.* Effect of Ovariohysterectomy at the Time of Tumor Removal in Dogs with Mammary Carcinomas: A Randomized Controlled Trial. *J. Vet. Intern. Med.* **30**, 230–241 (2016).

151. Cemazar, M. *et al.* Efficacy and safety of electrochemotherapy combined with peritumoral IL-12 gene electrotransfer of canine mast cell tumours. *Vet Comp Oncol* **15**, 641–654 (2017).

152. Saba, C. *et al.* A Comparative Oncology Study of Iniparib Defines Its Pharmacokinetic Profile and Biological Activity in a Naturally-Occurring Canine Cancer Model. *PLoS ONE* **11**, e0149194 (2016).

153. Wang, S.-L., Lee, J.-J. & Liao, A. T. Comparison of efficacy and toxicity of doxorubicin and mitoxantrone in combination chemotherapy for canine lymphoma. *Can. Vet. J.* **57**, 271–276 (2016).

154. Pellin, M. A. *et al.* Safety evaluation of combination doxorubicin and toceranib phosphate (Palladia®) in tumour bearing dogs: a phase I dose-finding study. *Vet Comp Oncol* **15**, 919–931 (2017).

155. Knapp, D. W. *et al.* A Nonselective Cyclooxygenase Inhibitor Enhances the Activity of Vinblastine in a Naturally-Occurring Canine Model of Invasive Urothelial Carcinoma. *Bladder Cancer* **2**, 241–250 (2016).

156. Harrington, B. K. *et al.* Preclinical Evaluation of the Novel BTK Inhibitor Acalabrutinib in Canine Models of B-Cell Non-Hodgkin Lymphoma. *PLoS ONE* **11**, e0159607 (2016).

157. Childress, M. O., Ramos-Vara, J. A. & Ruple, A. A randomized controlled trial of the effect of prednisone omission from a multidrug chemotherapy protocol on treatment outcome in dogs with peripheral nodal lymphomas. *J. Am. Vet. Med. Assoc.* **249**, 1067–1078 (2016).

158. Wouda, R. M., Hocker, S. E. & Higginbotham, M. L. Safety evaluation of combination carboplatin and toceranib phosphate (Palladia) in tumour-bearing dogs: A phase I dose finding study. *Vet Comp Oncol* **16**, E52–E60 (2018).

159. Saba, C. F. *et al.* Rabacfosadine for relapsed canine B-cell lymphoma: Efficacy and adverse event profiles of 2 different doses. *Vet Comp Oncol* **16**, E76–E82 (2018).

160. Keyerleber, M. A. & Ferrer, L. Effect of prophylactic cefalexin treatment on the development of bacterial infection in acute radiation-induced dermatitis in dogs: a blinded randomized controlled prospective clinical trial. *Vet. Dermatol.* **29**, 37-e18 (2018).

161. Sanchez, A. *et al.* Antihistaminic and cardiorespiratory effects of diphenhydramine hydrochloride in anesthetized dogs undergoing excision of mast cell tumors. *J. Am. Vet. Med. Assoc.* **251**, 804–813 (2017).

162. Bhat, A. H., Ganguly, B., Tiwari, A. K. & Das, A. K. Canine Parvovirus ns1 gene and Chicken Anemia vp3 gene induce partial oncolysis of Canine Transmissible Venereal Tumor. *Sci Rep* **7**, 15419 (2017).

163. Weishaar, K. M. *et al.* c-Kit Mutation and Localization Status as Response Predictors in Mast Cell Tumors in Dogs Treated with Prednisone and Toceranib or Vinblastine. *J. Vet. Intern. Med.* **32**, 394–405 (2018).
